# *MCAT* Mutations Cause Nuclear LHON-like Optic Neuropathy

**DOI:** 10.3390/genes12040521

**Published:** 2021-04-02

**Authors:** Sylvie Gerber, Christophe Orssaud, Josseline Kaplan, Catrine Johansson, Jean-Michel Rozet

**Affiliations:** 1 Laboratory of Genetics in Ophthalmology (LGO), INSERM UMR1163, Institute of Genetic Diseases, Imagine and Paris Descartes University, 75015 Paris, France; sylvie.gerber@inserm.fr (S.G.); josseline.kaplan@inserm.fr (J.K.); 2 Unité Ophtalmologie, Hôpital Européen Georges-Pompidou (HEGP), and Centre de Référence des Maladies Rares en Ophtalmologie (OPHTARA), Service d’Ophtalmologie, Hôpital Necker–Enfants Malades, 75015 Paris, France; christophe.orssaud@aphp.fr; 3 Botnar Research Centre, Nuffield Orthopaedic Centre, Headington, University of Oxford, Oxford OX3 7LD, UK; catrine.johansson@ndorms.ox.ac.uk

**Keywords:** hereditary optic neuropathy (HON), nuclear LHON-like, *MCAT*

## Abstract

Pathological variants in the nuclear malonyl-CoA-acyl carrier protein transacylase (*MCAT*) gene, which encodes a mitochondrial protein involved in fatty-acid biogenesis, have been reported in two siblings from China affected by insidious optic nerve degeneration in childhood, leading to blindness in the first decade of life. After analysing 51 families with negative molecular diagnostic tests, from a cohort of 200 families with hereditary optic neuropathy (HON), we identified two novel *MCAT* mutations in a female patient who presented with acute, sudden, bilateral, yet asymmetric, central visual loss at the age of 20. This presentation is consistent with a Leber hereditary optic neuropathy (LHON)-like phenotype, whose existence and association with *NDUFS2* and *DNAJC30* has only recently been described. Our findings reveal a wider phenotypic presentation of *MCAT* mutations, and a greater genetic heterogeneity of nuclear LHON-like phenotypes. Although *MCAT* pathological variants are very uncommon, this gene should be investigated in HON patients, irrespective of disease presentation.

## 1. Introduction

Hereditary optic neuropathies (HONs) are a group of degenerative disorders of the retinal ganglion cells, leading to optic nerve atrophy and visual loss. Their prevalence is ≥1:10,000, and they can be syndromic or not. The most frequent nonsyndromic HONs are maternally inherited Leber HON (LHON, MIM #535000) and autosomal dominant ON (ADON) or Kjer’s disease (MIM #165500). LHON is caused by mitochondrial DNA mutations in complex I or III subunits of the respiratory chain [1], and 60% to 90% of ADONs are caused by mutations in *OPA1*, which encodes a GTPase of the dynamin family that controls mitochondrial fusion [2,3] (MIM *605290). ADON is also associated with mutations in two other genes as follows, each accounting for no more than one or a few cases: *SPG7* [4], encoding paraplegin, a component of mitochondrial AAA proteases essential for OPA1 processing, and *DNM1L*, encoding another GTPase of the dynamin family involved in mitochondrial fission [5,6].

Nonsyndromic autosomal recessive ONs (ARONs) were initially described as very uncommon congenital severe HONs. However, by the early 2000s, it was clear that the frequency of the recessive forms had been largely underestimated and that they displayed substantial clinical variability—from congenital severe to late-onset slowly progressive ON—and genetic heterogeneity. One locus (*OPA6*) [7] and five disease-causing genes have been identified—namely, *TMEM126A* [8], *ACO2* [9], *RTN4IP1* [10], *WFS1* [11], and *NDUFS2* [12], respectively, encoding an inner mitochondrial membrane protein involved in complex I assembly [13], mitochondrial aconitase (MIM *100850), a mitochondrial ubiquinol oxidoreductase (RTN4-interacting protein 1, MIM *610502), an ER protein involved in ER–mitochondria interactions [14] (MIM *606201), and the 49-kDa complex I subunit (MIM *602985). A homozygous mutation in yet another gene encoding a mitochondrial protein—malonyl-CoA-acyl carrier protein transacylase (*MCAT*, MIM *614719), whose gene product is involved in fatty-acid biogenesis—has recently been identified in two brothers with early-onset, insidious ON, born to consanguineous Chinese parents [15].

We used Sanger sequencing of *MCAT* exon and intron–exon boundaries in families unresolved by HON gene panel screening, and identified new compound heterozygous mutations in a sporadic LHON-like case which differ strikingly from the initial description, expanding the spectrum of *MCAT* ocular presentations.

## 2. Materials and Methods

### 2.1. Families

We selected 51 families with a negative molecular diagnosis following targeted exome sequencing of mitochondrial DNA and a panel of 384 genes, including genes known to be associated with mitochondrial disorders and genes for complex I subunits and assembly factors. The cohort consisted of 15 sporadic cases (including 4 consanguineous), 24 simplex families, 10 multiplex families (including 2 with father-to-son transmission and 3 with maternal-line transmission), and 2 families with no pedigree information. This study was approved by the Comité de Protection des Personnes Ile-de-France II. Informed consent was obtained from each participant or legal representative.

### 2.2. MCAT Mutation Detection and Interpretation

We designed four primer pairs from the *MCAT* NM_173467.4 sequence to amplify the coding exons and flanking splice junctions of the gene as follows: (MCAT-1F) 5′-AAGCCCCGGCAGGAAATG-3′, (MCAT-1R) 5′-CGGAATGGCAGGCGGAAG-3′; (MCAT-2F) 5′-GGTACAGAGCCCTGGAAC-3′, (MCAT-2R) 5′-CACCTGAGTGGGTCCAGT-3′; (MCAT-3F) 5′-AGTGCAGAGTGGAGCTTG-3′, (MCAT-3R) 5′-CACGGGTGTGGAGCAGTT-3′; and (MCAT-4F) 5′-GCAGCTGTGGGTCTCTTT-3′, (MCAT-4R) 5′-CGACAGGCACAGCCTACA-3′. PCRs (10 µL) were performed under optimized conditions in Green GoTaq Flexi Buffer (1×; Promega, Charnonnières-les-Bains, France) containing the genomic DNA (50 ng) of probands, primer pairs (0.2 µM each), MgCl_2_ (1.5 mM), dNTP (0.04 mM) and GoTaq G2 Hot Start Polymerase (0.015 units; Promega). The PCR products were purified from single-strand DNA by adding exonuclease I (4 units; ThermoFisher, LifeTechnologies SAS, Villebon-sur-Yvette, France) and FastAP thermosensitive alkaline phosphatase (0.4 units; ThermoFisher), and incubated at 37 °C for 10 min. The reactions were stopped by heating the samples at 85 °C for 10 min. Sequencing reactions (10 µL) were performed on purified PCR products (2.5 µl) using forward or reverse primers (0.2 µM) and the BigDye Terminator v3.1 Cycle Sequencing Kit (Applied Biosystems, LifeTechnologies SAS, Villebon-sur-Yvette, France), in accordance with the manufacturer recommendations. The sequencing products were purified by spin column exclusion chromatography with a Sephadex G-50 and analysed with an ABI 3500XL genetic analyser.

Common polymorphisms were excluded based on frequencies extracted from the Genome Aggregation Database (gnomAD; https://gnomad.broadinstitute.org/), which includes both exome and genome sequencing data from a wide variety of large-scale sequencing projects. The consequences of rare variants (minor allele frequency of ≤1%) were predicted using the Alamut Visual gene browser’s Polyphen-2, SIFT, MutationTaster, NNSPLICE, MaxEntScan, and SpliceSiteFinder features (https://www.interactive-biosoftware.com/alamut-visual/) and the SpliceAI tool, which applies deep learning to identify splice variants (https://github.com/Illumina/SpliceAI) [16].

### 2.3. RNA Preparation, cDNA Synthesis, and RT-PCR Analysis

Total RNA was prepared from circulating blood leukocytes using the RNeasy Mini Kit (QIAGEN, Les Ulysses, France) in accordance with the manufacturer’s protocol. The samples were DNase-treated with the RNase-free DNase Set (QIAGEN). The concentration was assessed using the NanoDrop spectrophotometer (ThermoFisher) before storage at −80 °C. First-strand cDNA synthesis was performed from total RNA (500 ng) using the Verso cDNA Kit (ThermoFisher Scientific) with random hexamer:anchored oligo (dT) primers in a 3:1 (vol:vol) ratio, in accordance with the manufacturer’s instructions. A non-RT reaction (without an enzyme) for one sample was prepared as a control. *MCAT* cDNA was amplified by PCR using an oligonucleotide located in the 5’UTR region of the gene MCAT-F 5′-CCTCGGTCGCCACGGTA-3′, and the MCAT-4R primer (0.2 µM each) as described above. RT-PCR products were separated by electrophoresis in a 1.5% agarose gel stained with ethidium bromide, viewed under UV light, and sequenced using the Sanger method.

## 3. Results

**Figure 1:**
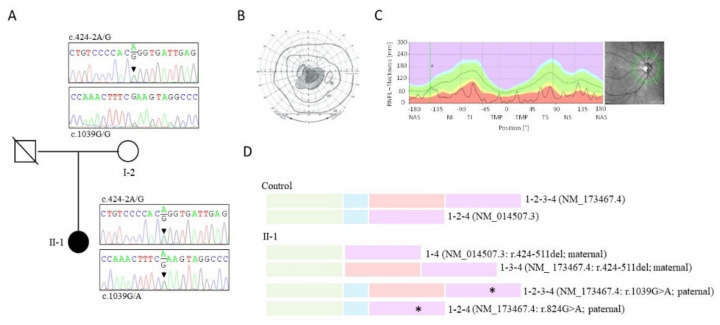
Pedigree, ophthalmologic, and genetic data of the Leber hereditary optic neuropathy (LHON)-like malonyl-CoA-acyl carrier protein transacylase (*MCAT*) case II-1 as follows: (**A**) Pedigree of the family and electropherograms of exons 2 and 4 showing compound heterozygosity for the NM_173467.4/NM_014507.3:c.424-2A>G and NM_173467.4:c.1039G>A (NM_014507.3:c.824G>A) pathological variants in the affected individual (II-1) and single heterozygosity for the c.424-2A>G mutation in her mother (I-2). The father died before the study. (**B**) Visual field (right eye) showing central scotoma. (**C**) Optic coherence tomography of the retinal nerve fibre layer (RNFL) showing marked thinning of the temporal and nasal RNFL. Solid line = II-1; dotted line = controls. Green area = normal RNFL thickness; red area = thickness of abnormally thin RNFL. NAS = nasal; NI = inferior nasal; TI = inferior temporal; TS = superior temporal; NS = superior nasal. (**D**) Schematic representation of the two *MCAT* splicing isoforms (with and without exon 3) transcribed from the *MCAT* gene in the controls, and the four isoforms transcribed in affected individual II-1 from the maternal and paternal alleles, respectively. Each *MCAT* exon is represented by a colour (green = exon 1; blue = exon 2; red = exon 3; purple = exon 4). The asterisk (*) indicates the paternal mutation in exon 4 (NM_173467.4:c.1039G>A, p.Glu347Lys; NM_014507.3:c.824G>A, p.Glu275Lys). Note that the maternal allele is transcribed into two splicing isoforms lacking exon 23.1.

Sanger screening of the four exons and intron–exon junctions of *MCAT*, in 51 HON families with negative molecular diagnoses, led to the identification of compound heterozygous mutations in one individual (II-1; Figure 1A).

### 3.1. Clinical Findings

Subject II-1 is the only child of unrelated parents of French origin with no history of visual deficiency. She was born after an uneventful pregnancy and had no health issues. Twenty years ago, on her 20th birthday, she experienced a sudden bilateral, yet asymmetrical (predominantly affecting the left eye), decrease in visual acuity together with headaches. Two months later, her visual acuity had dropped from 10/10 for both her left and right eyes—i.e., normal—to 1/10 on the right and <1/10 on the left, and she exhibited a central scotoma. The electroretinograms were normal, but the visual evoked potentials were altered. The clinical record mentions a brain MRI at that time, showing optic nerve hypersignals extending to the chiasma, particularly on the left side. Cerebrospinal fluid analysis and neurological examination were unremarkable as were infectious and cardiac workups. The patient was admitted to the hospital to receive boluses of methylprednisolone (solumedrol; 1 g/day for three days) but experienced further loss of the left-eye visual performance (poorly oriented light perception) while being treated. Ten days later, she received three more boluses of methylprednisolone, which did not improve her visual acuity. LHON was suggested as a diagnosis, but mitochondrial DNA sequencing turned up no disease-causing mutation other than the NC_012920.1:m.15257G>A (YP_003024038.1:p.Asp171Asn) variant of the cytochrome b gene, deemed a secondary mutation insufficient to cause disease. A decade later, the patient was seen at the French national reference centre for rare ophthalmologic diseases (OPHTARA). Her vision had dropped to 1/20 and 1/40 for the left and right eyes, respectively, and she exhibited a central scotoma (Figure 1B)—but normal peripheral vision—as well as photophobia and dyschromatopsia (blue–yellow axis). Retinal nerve fibre layer optical coherence tomography revealed a dramatic generalized reduction in optic nerve fibres (Figure 1C).

### 3.2. Molecular Findings

*MCAT* has four coding exons and is transcribed into two isoforms by the alternative splicing of exon 3. The splicing isoforms composed of exons 1 to 4 (NM_173467.4) and exons 1, 2, and 4 (NM_014507.3) (Figure 1D) encode proteins having 391 and 181 amino acids, respectively. *MCAT* screening identified an unreferenced splice donor site variant NM_173467.4/NM_014507.3:c.424-2A>G (intron 1), and an ultra-rare missense substitution NM_173467.4:c.1039G>A, p.Glu347Lys (NM_014507.3:c.824G>A, p.Glu275Lys; rs375520104; a minor allele frequency of 0.0053%) (Figure 1A). Segregation analysis showed maternal transmission of the missense mutation, suggesting biallelism of the two *MCAT* changes (the paternal DNA is unavailable; Figure 1A).

In silico analysis of the NM_173467.4/NM_014507.3:c.424-2A>G variant predicted the complete abolition of the intron 1 splice donor site, and the skipping of exon 2 (Figure S1). According to the Amercian College of Medical Genetic and Genomics (ACMG standards and guidelines [17], this variant is predicted to be pathogenic (PVS1). The analysis of leukocyte mRNA from the controls identified the NM_173467.4 and NM_014507.3 isoforms (Figure 1D and Figure S3A). NM_173467.4 (exons 1 to 4) mRNA was less abundant than NM_014507.3 (exons 1, 2, and 4) mRNA, suggesting greater expression or preferential PCR amplification of the mRNA species with a lower molecular weight (Figure S3A). Reverse-transcribed leukocyte mRNAs from the patient were amplified using forward and reverse primers located in exon 1 and in exon 4 or 3, respectively. Agarose gel analysis and Sanger sequencing of RT-PCR products detected the splicing isoforms composed of exons 1 to 4 (NM_173467.4) or exons 1, 2, and 4 (NM_014507.3) arising from the paternal allele, and shorter mRNAs composed of exons 1, 3, and 4 (NM_173467.4:r.424-511del), or exons 1 and 4 (NM_014507.3: r.424-511del) transcribed from the maternal allele carrying the c.424-2A>G splicing-site mutation (Figure 1D and Figure S2). We note that the mRNA isoforms consisting of exons 1 to 4 and exons 1, 3, and 4, respectively, were detected when the reverse-transcribed mRNA was amplified using the reverse primer located in exon 3 but not exon 4 (Figure S2A–B), suggesting higher PCR competition in patient leukocytes expressing the low-molecular-weight product composed of exons 1 and 4, which is not found in the controls.

The second pathological variant carried by the female patient—NM_173467.4 c.1039G>A, p.Glu347Lys (NM_014507.3:c.824G>A, p.Glu275Lys)—affects a residue which is evolutionarily conserved through *Escherichia coli* (phyloP: 7.52 (−20.0;10); Figure S3), strongly suggesting an important functional role. Its substitution for a lysine is predicted to be deleterious by SIFT (score: 0; median: 3.09), disease-causing by MutationTaster (prob: 1), and probably damaging by Polyphen-2 (score: 1). According to the ACMG standards and guidelines, this variant is predicted as likely pathogenic (detected in trans with a PVS1 variant (PM3), an extremely low frequency in controls (PM2), multiple lines of computational evidence supporting a deleterious effect on the gene and the gene product (PP3), and the patient’s phenotype is consistent with mutations in the gene (PP4)).

### 3.3. In Silico Analysis of the Effect of MCAT Mutations on the 3D-Structure of the Enzyme

**Figure 2:**
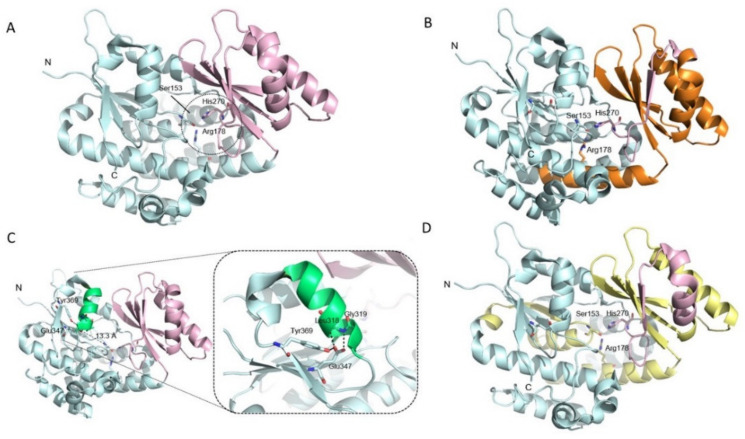
Structure of human MCAT isoforms and mutant products. The MCAT structure cartoon was prepared with Protein Data Bank (PDB) coordinates for 2C2N (https://www.rcsb.org/) using the PyMOL molecular visualization system, version 1.7.4.0 (Schrödinger, LLC, New York, NY, USA). (**A**) The full-length protein is composed of a large domain (cyan) and a smaller one (pink). The active-site residues, Ser153, His270, and Arg178, are shown in a gorge between the two subdomains. (**B**) The protein isoform lacking the amino acid sequence encoded by exon 3 (orange) lacks most of the smaller domain and Arg178, and it is likely inactive. (**C**) A full and zoom view of Glu347 in the structure. Glu347 is 13.3 Å away from the active-site Ser153. It interacts electrostatically with the side chain of Tyr369 and the backbone amides of Leu318 and Gly319, stabilizing a helix formed by Arg352–Cys361 (green) at the interface between the two domains. (**D**) The isoform missing residues 142 to 243 (yellow) lacks almost all of the smaller domain, the active-site residues Ser153 and Arg178, and elements of the secondary structure important to the activity of the enzyme.

The MCAT isoform produced from the mRNA with exons 1 to 4 (NM_173467.4) has two differently sized domains. The active-site residues, Ser153, His270 and Arg178, are located in a gorge between the two subdomains (Figure 2A). His270 contributes to the nucleophilicity of the active-site Ser153, and Arg178 aids in the positioning of the malonate 3-carboxyl of the malonyl-CoA substrate. The mutation of Arg178 into Gln, Gly, or Ala has been shown to reduce the activity of the enzyme and its affinity for the malonyl substrate [18]. The MCAT product arising from the mRNA with exons 1, 2, and 4 (NM_014507.3), lacks most of the smaller domain and Arg178, and it is most likely inactive (Figure 2B). We examined the effect that glutamic acid substitution, through the paternal mutation c.1039G>A, might have on the 3D structure of the full-length MCAT isoforms. Glu347 interacts electrostatically with the side chain of Tyr369 and the backbone amides of Leu318 and Gly319, stabilizing a helix formed by Arg352–Cys361 at the interface between the two domains (Figure 2C). Thus, a mutation of the negatively charged Glu347 to the positively charged lysine will disrupt these interactions and shift the helix, which may affect interactions with the substrate and the interacting proteins. 

The mutant mRNA isoform composed of exons 1, 3, and 4, transcribed from the maternal allele carrying the c.424-2A>G substitution (Figure 1D), encodes a premature termination codon (p.Ile143Cysfs*4). Even if the mRNA was translated, the product would encompass the first two or three helices (one being only partly formed) and a beta-strand in the larger domain but miss both the active-site and the substrate- binding pocket. The protein would likely be unstable as the beta-strand would not be packed against the surrounding helices. In contrast, the second mutant isoform transcribed from the maternal allele, which lacks exons 2 and 3, contains an open reading frame. The translation of the mRNA composed of exons 1 and 4 would produce a 289-amino-acid protein lacking the 101 central residues of the full-length MCAT protein (p.Val142_Glu243del). The deletion of residues 142 to 243 would nearly delete the smaller domain, as well as several beta-strands and alpha-helices in the larger domain that harbour the active-site residues Ser153 and Arg178 (Figure 2D). It would therefore have a substantially detrimental effect on the protein, which would very possibly be inactive.

## 4. Discussion

We reported on the identification of biallelic mutations in the malonyl-CoA-acyl carrier protein transacylase which cause nonsyndromic LHON-like optic neuropathy in a sporadic case concerning an adult female. This is the second report of mutations in this gene, which contributes to fatty-acid biogenesis in the mitochondria. The initial report described two brothers who were born to consanguineous parents from China and carried two homozygous missense mutations, one of which (p.Leu81Arg) had a prominent effect on protein stability and mitochondrial inner membrane morphology [15]. The two siblings were described as suffering from gradual, insidious vision loss leading to severe visual dysfunction, nystagmus, and visual acuity reduced to finger counting by the age of 8—a phenotype that sharply contrasts with the disease presentation we report here: acute, bilateral yet asymmetric visual loss in early adulthood, progressing quickly (<2 months) to central blindness. This distinctive mode of onset may be due to a genotypic difference in *MCAT* or the mitochondrial DNA sequence. This raises the question of the contribution of the m.15257G>A (p.Asp171Asn) variant, which alters a highly conserved amino acid in an extra-membrane domain of cytochrome b. There has been some controversy about the significance of the 15257 variant—found also at a low frequency in control populations—because LHON in individuals with the substitution may actually be the result of other mutations they carry [19,20,21,22,23]. Furthermore, the ClinVar database (SCV000998307.1) deems it to be a benign variant, according to the updated recommendation for the benign stand-alone ACMG/AMP criterion (BA1: variant whose minor allele frequency is >0.05 is considered benign). Whether the m.15257G>A substitution contributes to the disease presentation in the patient is open to debate. The mode of onset is highly reminiscent of LHON, as is retinal vascular tortuosity. We very recently disclosed the existence of LHON-like diseases ascribed to pathological variants in the nuclear genes *NDUFS2* [12] and *DNAJC30* [24]. Individuals carrying recessive variants in these genes displayed nonhemorrhagic papillary pseudo-oedema or papillary pseudo-oedema with peripapillary telangiectasia [12,24]. Images of the retinal fundus of the patient in the acute phase of the disease were not available to study these symptoms, which are pathognomonic features of LHON [25].

Nonsyndromic optic neuropathies have been ascribed to sequence variations in genes including *SPG7* (MIM *602783), *WFS1* (MIM *606201), *ACO2* (MIM *100850), *NDUFS2* (MIM *602985) and *DNM1L* (MIM *603850), severe mutations of which have reportedly caused the systemic mitochondrial symptoms associated with spastic paraplegia, Wolfram syndrome, infantile cerebellar-retinal degeneration, mitochondrial complex I deficiency, and lethal encephalopathy, respectively. This is equally true for *OPA1*, LHON genes, and *TMEM126*-related HON, where silent to severe (“plus”) neurological, cardiac, muscular, and auditory symptoms have been described [1,26,27], as well as for RTN4IP1, whose initial involvement in HON was secondarily extended to early-onset severe encephalopathies [28]. These observations, which suggest that HONs are an endophenotype of mitochondrial cytopathies [5], raises the question of the *MCAT*-null phenotype, given that neither the two Chinese brothers initially reported nor the patient we present here are thought to carry fully inactivating mutations. The question is all the more valid as the *Mcat* conditional knockout mouse reportedly exhibits reduced muscle strength, kyphosis, hypothermia, and a shortened lifespan, attributed primarily to reduced mitochondrial respiration [29].

## 5. Conclusions

The case presented here expands on the range of ocular presentations of pathological *MCAT* variants, which should be systematically investigated in HON, regardless of age and mode of onset of vision loss. Our report also suggests greater genetic heterogeneity underlying nuclear LHON-like phenotypes, which may be more common than previously thought.

## Supplementary Materials

The following are available online at https://www.mdpi.com/article/10.3390/genes12040521/s1: Figure S1: *MCAT* intron 1/exon 2 regions encompassing the pathological c.424-2A>G variant, and splicing score predictions; Figure S2: RT-PCR and Sanger sequencing analysis of leukocyte mRNA from the control, and index case II-1; Figure S3: alignment of the MCAT sequence showing strict conservation of glutamic acid 347 across species, from human to bacteria.
