# Supplementary material for: MCAT Mutations Cause Nuclear LHON-like Optic Neuropathy
Source: Genes (Basel). 2021 Apr 2;12(4):521. doi: 10.3390/genes12040521 (PMC8067165; doi:10.3390/genes12040521)
Supplement: Supplementary file 1 [file genes-12-00521-s001.pdf]

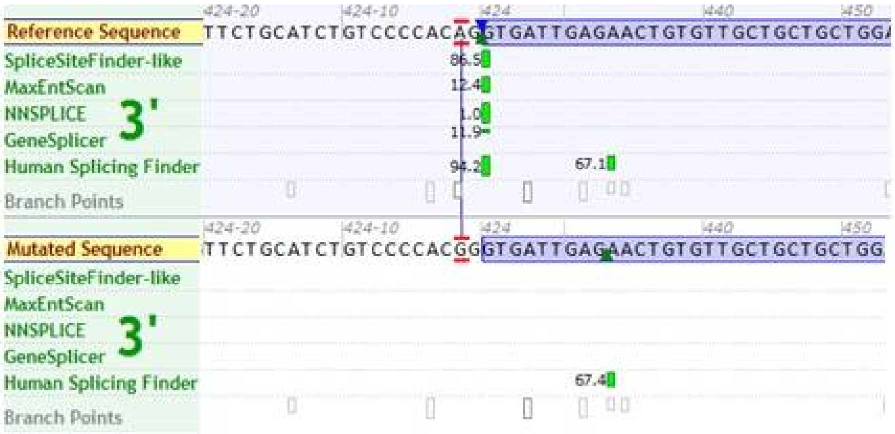

Supplementary Figure 1. *MCAT* intron 1 / exon 2 region encompassing pathological c.424-2A>G variant, and splicing score predictions. The consensus splice site predicted in the wildtype sequence (reference sequence) is lost in the sequence harbouring the c.424-2A>G change (Mutated sequence), according to the five prediction programs available through the Alamut software. This is predicted to result in the skipping of exon 2.

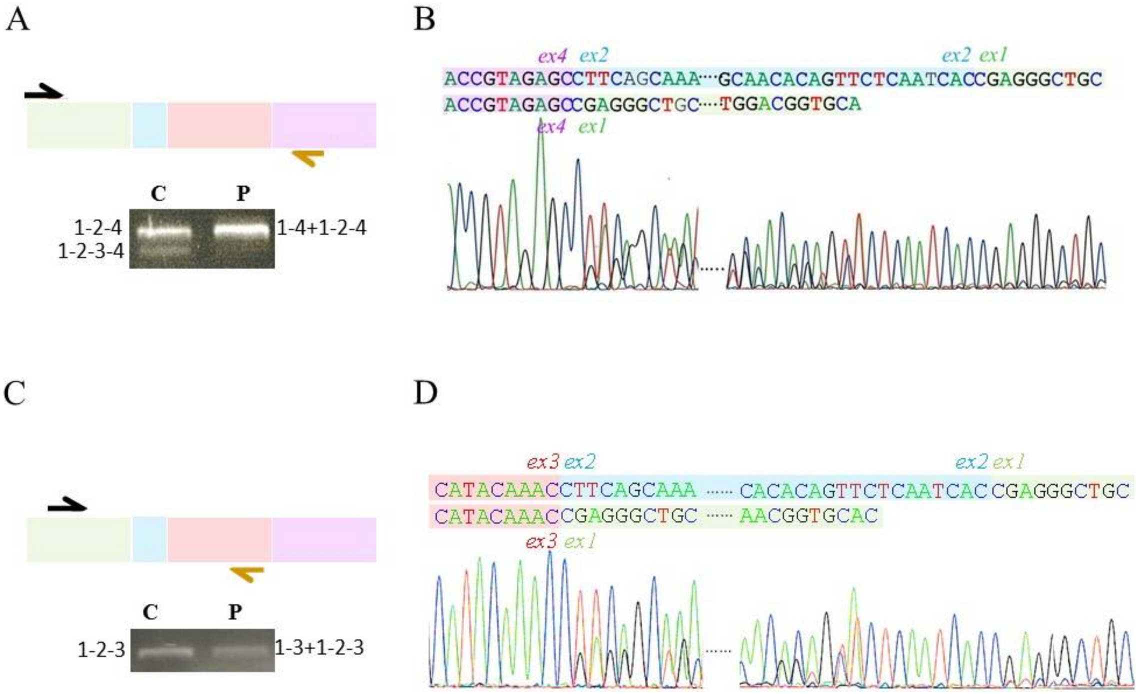

Supplementary Figure 2. RT-PCR and Sanger sequencing analysis of leukocyte mRNA from control and index case II-1. Simplified representations of four *MCAT* exons (green = exon 1; blue = exon 2; red = exon 3; purple = exon 4) and agarose gel images showing cDNA products amplified using forward and reverse primers (arrows) in **(a)** exons 1 and 4 and **(c)** exon 1 and 3 (C = control; P = case II-1). Exonic composition of cDNA products amplified from II-1 leukocytes was determined by Sanger sequencing using reverse primers (see gold arrows in left panels) in **(b)** exon 4 and **(d)** exon 3. Sequence electropherograms obtained from control cells are not shown.

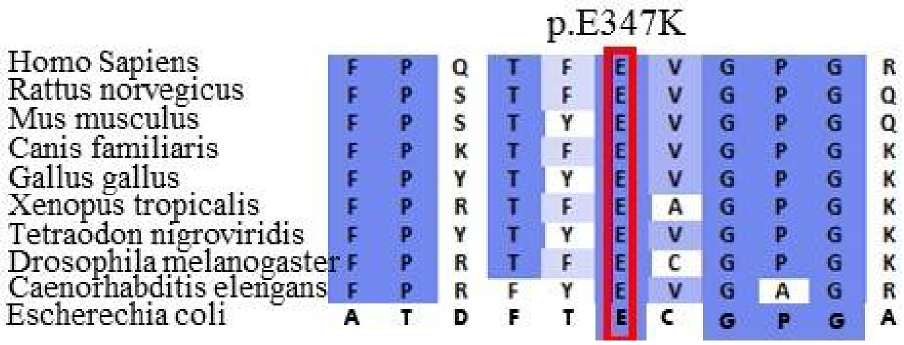

Supplementary Figure 3. Alignment of MCAT sequence showing strict conservation of glutamic acid 347 across species, from human to bacteria. Numbering of residues refers to NM_173467.4.
